# Programmable patterned MoS_2_ film by direct laser writing for health-related signals monitoring

**DOI:** 10.1016/j.isci.2021.103313

**Published:** 2021-10-16

**Authors:** Manzhang Xu, Jiuwei Gao, Juncai Song, Hanxin Wang, Lu Zheng, Yuan Wei, Yongmin He, Xuewen Wang, Wei Huang

**Affiliations:** 1 Frontiers Science Center for Flexible Electronics and Xi'an Institute of Flexible Electronics (IFE), Northwestern Polytechnical University, Xi'an 71002, P. R. China; 2 State Key Laboratory of Chemo/Biosensing and Chemometrics, College of Chemistry and Chemical Engineering, Hunan University, Changsha 410082, P. R. China; 3 MIIT Key Laboratory of Flexible Electronics (KLoFE), Northwestern Polytechnical University, Xi'an 710072, P. R. China; 4 Shaanxi Key Laboratory of Flexible Electronics (KLoFE), Northwestern Polytechnical University, Xi'an 710072, P. R. China; 5 State Key Laboratory of Organic Electronics and Information Displays, Institute of Advanced Materials (IAM), Nanjing University of Posts & Telecommunications, Nanjing 210023, P. R. China; 6 Key Laboratory of Flexible Electronics (KLoFE) and Institute of Advanced Materials (IAM), Nanjing Tech University, Nanjing 211800, P. R. China

**Keywords:** Physics, Sensor system, Nanomaterials

## Abstract

The two-dimensional (2D) transition metal dichalcogenides (TMDs) are promising flexible electronic materials for strategic flexible information devices. Large-area and high-quality patterned materials were usually required by flexible electronics due to the limitation from the process of manufacturing and integration. However, the synthesis of large-area patterned 2D TMDs with high quality is difficult. Here, an efficient and powerful pulsed laser has been developed to synthesize wafer-scale MoS_2_. The flexible strain sensor was fabricated using MoS_2_ and showed high performance of low detection limit (0.09%), high gauge factor (1,118), and high stability (1,000 cycles). Besides, we demonstrated its applications in real-time monitoring of health-related physiological signals such as radial artery pressure, respiratory rate, and vocal cord vibration. Our findings suggest that the laser-assisted method is effective and capable of synthesizing wafer-scale 2D TMDs, which opens new opportunities for the next flexible electronic devices and wearable health monitoring.

## Highlights

• Wafer-scale patterned MoS_2_ film has been synthesized by pulsed laser
• The MoS_2_ film strain sensor shows low limit detection, high GF, and stability
• The healthy-related singles have been monitored by the MoS_2_ film strain sensor

## Graphical abstract

**Figure:**
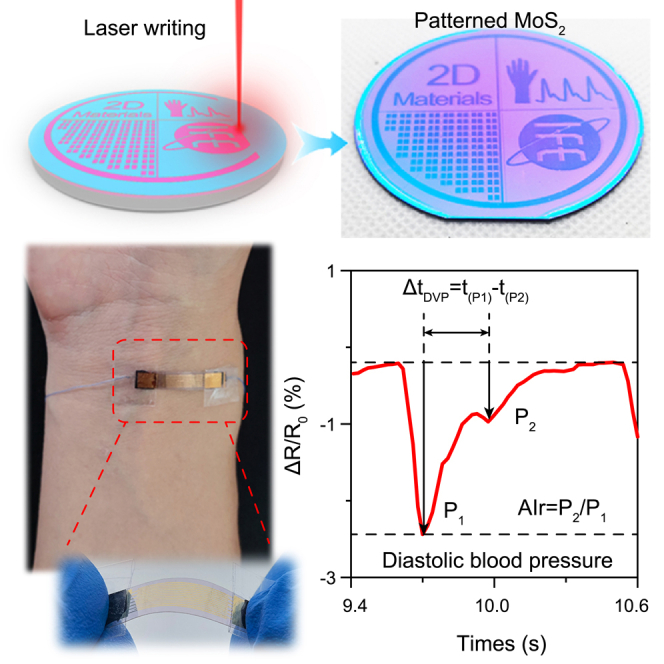

## Introduction

Flexible electronic sensors are the essential components of a new generation of strategic flexible information devices. As the basic and widely used devices, flexible strain sensors have attracted increasing attention (Amjadi et al., 2016; An et al., 2001; Liu et al., 2021; Wang et al., 2020c). High-quality, flexible electronic materials are the core of high-performance flexible electronic sensors, and the development of novel electronic materials has extensively promoted the development of flexible electronic technology (He et al., 2019; Sun et al., 2019; Wang et al., 2020b, 2021; Yan et al., 2021). As burgeoning flexible electronic materials, two-dimensional (2D) materials, especially transition metal dichalcogenides (TMDs), have been widely studied due to their unique physical and chemical properties that occur due to their atomic thickness (Jiang et al., 2020; Wang et al., 2020a; Zheng et al., 2021; Zhu et al., 2021). However, flexible electronic devices usually require large areas and high-quality materials; the process of manufacture and integration restricts their applications (Kim et al., 2015; Leong, 2020; Yu et al., 2020).

Controllable synthesis of wafer-scale TMDs is the pursuit of scientific researchers (Kim et al., 2021; Tong et al., 2019). Recently, the chemical vapor deposition (CVD) method has shown enormous potential for synthesizing high-quality crystalline 2D materials (Yang et al., 2020; Yu et al., 2017). However, additional patterning process is essential when 2D TMD films are used for devices fabrication. The patterning technologies for 2D materials, such as laser lithography, plasma etching, and photolithography, increase device fabrication complexity and negate some of the advantages of the CVD method. Therefore, achieving high-precision, programmable, patterned wafer-scale 2D TMDs is still a challenge. Recently, lasers have been used to synthesize materials because they minimize the thermal effect, have high manufacturing precision, and have fast preparation speed (Hu et al., 2020; Jung et al., 2019; Lu et al., 2014; Park et al., 2020a, 2020b). In addition, programmable patterns can be obtained in one step using a maskless laser and photolithography to avoid material contamination and reduce process flow (Cao et al., 2013; Mohapatra et al., 2020).

In this study, the laser-assisted method was used to synthesize programmable wafer-scale MoS_2_. Semitransparent flexible strain sensors were fabricated using the laser-written MoS_2_ film. The electromechanical behavior of the MoS_2_ film strain sensor was then investigated, and applications of the sensor were explored. Health-related signals have been monitored, such as radial artery pressure, respiratory rate, and vocal cord vibration.

## Results and discussion

### Direct laser synthesis of MoS_2_ film

**Figure 1:**
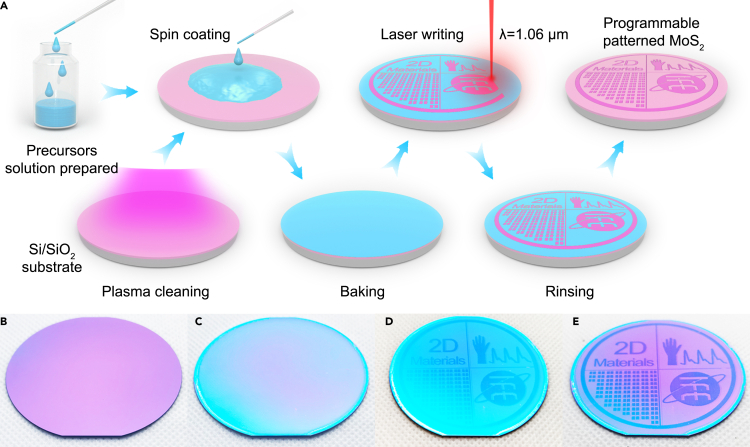
Direct laser writing of programmable patterned MoS_2_ film (A) Flow diagram of direct laser writing, a laser-directed synthesis of MoS_2_ on SiO_2_/Si wafer. (B–E) Photographs of the 2-inch SiO_2_/Si wafer after plasma cleaning, baking, laser writing, and rinsing process, respectively.

The programmable patterned MoS_2_ film process is illustrated in Figure 1A. A commercial laser marking machine equipped with a 1.06-μm pulsed fiber laser was used to synthesize the MoS_2_ film. Before laser writing, the SiO_2_/Si wafer with an oxidation layer of 285 nm was cleaned with plasma (Figure 1B). The ammonium tetrathiomolybdate ((NH_4_)_2_MoS_4_) precursor solutions were prepared and spin coated on the SiO_2_/Si wafer. Then, the SiO_2_/Si wafer was baked to evaporate residual solvents (Figure 1C). The laser writing patterns can be precisely designed using EZCAD software, as shown in Figure 1. The programmable pattern can be designed with text, lines, patterns, and matrices, and the pattern of the MoS_2_ film can be quickly finished in a few minutes. After laser writing, the color of the SiO_2_/Si wafer changed, indicating that the chemical reaction occurred in the laser-written area, as shown in Figure 1D. In addition, the location area reaction temperature was higher than the decomposition temperature of (NH_4_)_2_MoS_4_, and the following chemical reactions occurred (Park et al., 2020b; Sang et al., 2019).

(Equation 1) $${\left(NH_{4}\right)}_{2}MoS_{4}\overset{Laser}{\to }2NH_{3}+H_{2}S+MoS_{3}$$

(Equation 2) $$MoS_{3}\overset{Laser}{\to }S+MoS_{2}$$

After laser writing, the SiO_2_/Si substrate was rinsed in dimethylformamide solution (DMF) to remove any residual precursor film, and a programmable MoS_2_ pattern remained on the SiO_2_/Si substrate. As shown in Figure 1E, a programmable MoS_2_ film with a pattern of “2D Materials,” matrix electrodes, wrist and pulse waves, and the logo can be effectively and efficiently realized.

### Characterizations of MoS_2_ film

**Figure 2:**
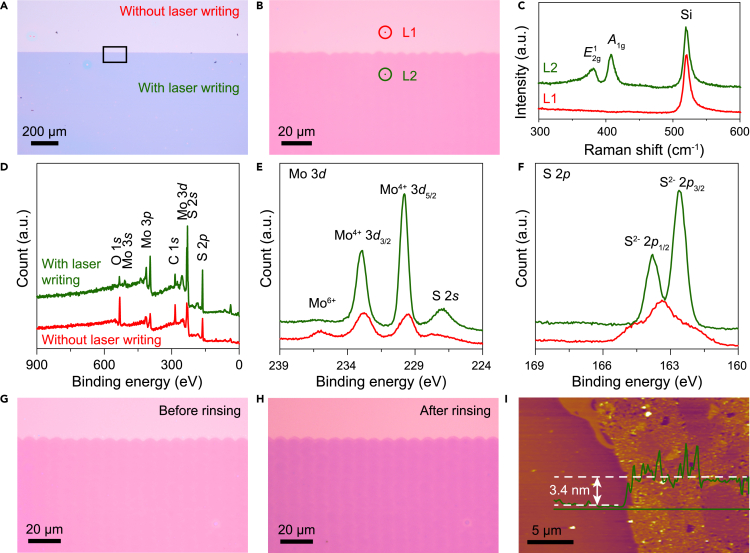
Characterization of the direct laser-written MoS_2_ film (A and B) Optical images of MoS_2_ film on SiO_2_/Si substrate after direct laser writing. The high-magnification optical image shown in (B) is taken from the black box shown in (A). (C) Raman spectrum of locations with and without laser writing obtained from the locations marked in (B). (D–F) XPS spectra of the film on SiO_2_/Si substrate with and without laser writing. (G and H) Optical images of MoS_2_ film on SiO_2_/Si substrate before and after rinsing. (I) AFM image of direct laser writing MoS_2_ film.

Optical image of the MoS_2_ film after laser writing was shown in Figure 2A; the change in color could also be identified by the eyes (Figure 1D). From the optical images, the continuous dense film could be successfully synthesized. High-magnification optical images are shown in Figure 2B. The semicircular wavy line, which was caused by the circular spot of the laser, divides the laser writing area. The *in situ* optical and scanning electron microscopic (SEM) images of the MoS_2_ films have been added in Figure S1. The low-magnification optical and corresponding SEM images in Figures S1A and S1B indicated that the MoS_2_ uniformity films had been successfully synthesized by laser. The high-magnification SEM image of the interface with and without laser writing is shown in Figure S1D. The very vague interface might be caused by the weak variation of the films with and without laser writing. The divisional interface semicircular wavy line is faintly visible, which indicates that the MoS_2_ films and precursor films are uniforms. The Raman spectrum was used to identify the materials, as shown in Figure 2C. The Raman spectrum showed a dramatic change in the laser writing area. Without laser writing, only one typical Raman peak, located at 520 cm^−1^, was consistent with the Raman peak of Si and raised from SiO_2_/Si substrate. After laser writing, apart from the Si peaks, two typical Raman peaks at 381.6 cm^−1^ and 407.1 cm^−1^ were observed, indicating that the 2D 2H phase MoS_2_ crystal film was successfully synthesized. The peak at 381.6 cm^−1^ $\left(E_{2g}^{1}\right)$ was attributed to the in-plane vibrational mode, and the peak at 407.1 cm^−1^ $\left(A_{1g}\right)$ was attributed to the out-of-plane vibrational mode (Li et al., 2012; Mohapatra et al., 2020). X-ray photoelectron spectroscopy (XPS) was used to investigate the variation further during laser writing, as shown in Figures 2D–2F. A comparison of the XPS full spectra of the film with and without laser writing is shown in Figure 2D. As shown, the intensity of Mo 3*d*, Mo 3*p*, S 2*p* was increased, which indicated that the surface element concentration of the Mo and S increased during laser writing. The high-resolution XPS spectra of Mo 3*d* and S 2*p* is shown in Figures 2E and 2F. Compared with the Mo 3*d* spectrum of the samples with and without laser writing (Figure 2E), four peaks were centered at 235.9, 232.7, 229.6, and 227.8 eV, which was consistent with peaks of Mo^6+^, Mo^4+^, Mo^4+^, and S 2*s*. The peaks indicated that Mo^6+^ was present in the film without laser writing (Park et al., 2020a). With laser writing, the intensity of the Mo^4+^ and peak S 2*s* increased, whereas the Mo^6+^ peak disappeared. The binding energy of Mo 3*d* was centered at 232.9 and 229.8 eV, which was consistent with Mo^4+^ 3*d*_3/2_ and Mo^4+^ 3*d*_5/2_, respectively (Fan et al., 2020; Park et al., 2020a). The binding energy of Mo^6+^ disappeared, which indicated that all Mo was reduced from Mo^6+^ to Mo^4+^ (Fan et al., 2020). Compared with the S 2*p* spectrum, in Figure 2F, the S 2*p* spin-spin split from the single peak (163.3 eV) into 2*p*_1/2_ (163.8 eV) and 2*p*_3/2_ (162.6 eV) with laser writing, which signified the formation of 2H-MoS_2_ (Hu et al., 2020; Park et al., 2020a). The unreacted precursor film was rinsed using DMF to obtain the thickness of the MoS_2_ film. The *in situ* optical images of the MoS_2_ film before and after rinsing are shown in Figures 2G and 2H, respectively. The atomic force microscopic (AFM) image of the MoS_2_ film is shown in Figure 2I. The MoS_2_ had a thickness of 3.4 nm and an average roughness (R_a_) of 0.78 nm, indicating that five layers of MoS_2_ were successfully synthesized.

### MoS_2_ film strain sensor fabrication and electromechanical behavior

**Figure 3:**
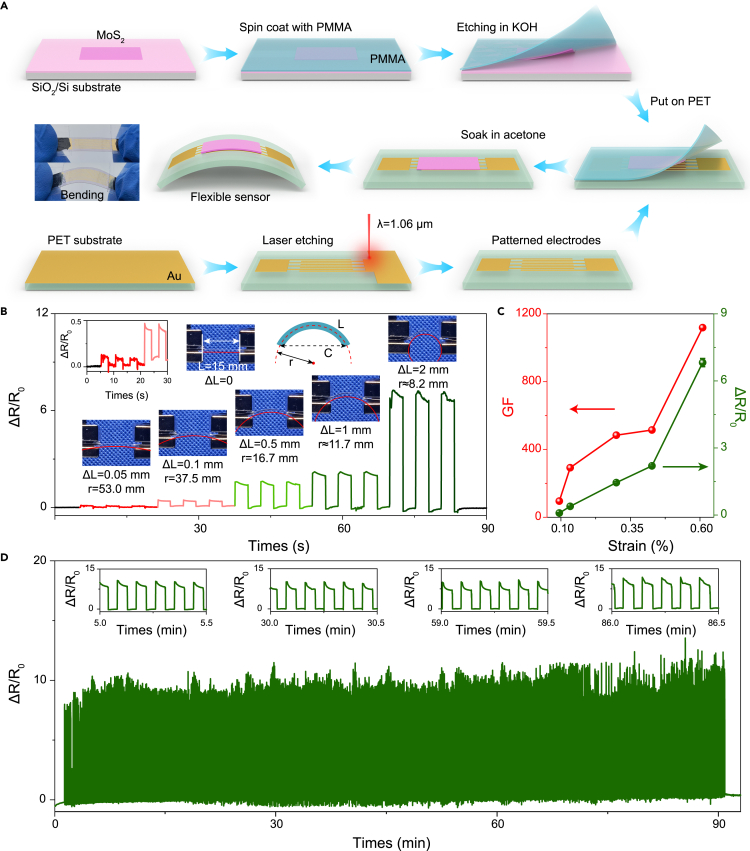
Fabrication of the flexible MoS_2_ film strain sensor and electromechanical behavior (A) Flow diagram of the fabrication of the flexible MoS_2_ film strain sensor. (B) The flexible MoS_2_ film strain sensor response under forward bending. The inset graph is the enlarged area of the sensor response from 0 to 30 s. The inset photographs were taken during the forward bending state under different ΔL. (C) The strain-dependent sensor response and GF. (D) Relative MoS_2_ film strain sensor response during 1,000 stretch and release cycles.

Owing to their atomic thickness, 2D material can withstand large deformations without breaking and exhibit high-performance strain sensing (Jiang et al., 2020; Liu et al., 2014; Park et al., 2016, 2019). Therefore, layered 2D materials, such as MoS_2_, have unique advantages in applications of flexible stress sensors. The MoS_2_ film strain sensor was fabricated as shown in Figure 3A. The MoS_2_ film was transferred from the SiO_2_/Si substrate using the polymethyl methacrylate (PMMA)-assisted transfer method. A 50-nm Au interdigital electrode that had an electrode spacing of 100 μm was fabricated using laser etching technology on the polyethylene terephthalate (PET) substrate. The MoS_2_ film covered the PET substrate, and PMMA was removed by acetone. The electromechanical behavior of the MoS_2_ film strain sensor is shown in Figures 3B–3D. The PET substrate bends while under different compressive lengths (ΔL), resulting in the resistance value of the MoS_2_ film strain sensor. The initial resistance (R_0_) of the MoS_2_ film strain sensor is 8.0 MΩ. Under the forward bending, the resistance of the MoS_2_ film strain sensor is increased. The sensor response can be calculated as 0.09, 0.39, 1.45, 2.20, and 6.82 under different compressive lengths (ΔL) of 0.05, 0.1, 0.5, 1, and 2 mm shown in Figure 3B. Under the reversed bending, the resistance of the MoS_2_ film strain sensor shows the opposite evolution result indicating that our MoS_2_ film strain sensor is the piezoresistive sensor (Figure S2). The sensor response can be calculated to be −0.4 under the reversed bending with ΔL of 1 mm. Because compressive stress causes PET to bend, the radius of curvature (r) can be quantified under the bending state of the MoS_2_ film on PET. As shown in Figure 3B, r is determined by chord length (*C*) and arc length (*L*) using the following formula (Liao et al., 2015):

(Equation 3) C=2rsin(L/2r)

The r can be calculated to be 53.0, 37.5, 16.7, 11.7, and 8.2 mm with ΔL increases from 0.05 mm to 2 mm. Thus, the bending strain (ε) can be calculated by the following formula (Lee et al., 2019; Zhu et al., 2013):

(Equation 4) $$\varepsilon =\frac{Z}{2r}$$

where Z is the thickness of the MoS_2_ film strain sensor (100 μm).

Therefore, the sensor responses of 0.09, 0.39, 1.45, 2.20, and 6.82 were measured under the increased strain of 0.09%, 0.13%, 0.29%, 0.43%, and 0.61%, respectively, as ΔL increased from 0.05 to 2 mm. The gauge factor (GF) of the MoS_2_ film strain sensor is calculated as follows (Liao et al., 2015; Manzeli et al., 2015; Pang et al., 2012):

(Equation 5) $$GF=\frac{\Delta R/R_{0}}{\varepsilon }$$

The strain-dependent sensor response (with standard deviation) and GF are shown in Figure 3C. The MoS_2_ film strain sensor effectively detected the small strain with a low detection limit of 0.09%. The GF reached 95 and 1,118 under strains of 0.09% and 0.61%. The low detection limit and GF were much higher than the numbers reported in the literature (Biccai et al., 2019; John et al., 2020; Manzeli et al., 2015; Neri and López-Suárez, 2018; Park et al., 2016, 2019, 2020a; Pei et al., 2020; Qiu et al., 2019; Rana et al., 2020; Tsai et al., 2015; Wu et al., 2014; Zheng et al., 2018; Zhu et al., 2019a, 2019b). In addition, the stability testing of the MoS_2_ film strain sensor was performed before it was used in practical applications. As shown in Figure 3D, a large strain (ε = 0.61%, r = 8.2 mm) was used to complete 1,000 stretching and releasing cycles. During this cycle test, the resistance changes of the baseline and peak remained stable. Four enlarged stretching and releasing cycles at 5 min, 30 min, 59 min, and 86 min were enlarged, and the results indicated that high stability of the MoS_2_ film strain sensor was achieved. The high GF and low detection limit mainly rise from the native nature of the high-quality MoS_2_ films synthesized with laser. On the one hand, MoS_2_ has an excellent strain performance. Owing to the piezoresistive effect, the band gap of 2D MoS_2_ decreases with the increase of strain. The direct band gap of monolayer TMDs changes to indirect band gap when the stain is 1%–2%, which significantly affects the sensor response under the small strain (Lee et al., 2019; Li et al., 2019; Qi et al., 2015; Tsai et al., 2015). On the other hand, with the laser writing, some minimal homogeneous cracks might exist in the MoS_2_ films. The reversible response of the MoS_2_ films is attributed to the homogeneous crack formation in the structure, which further promotes the high GF and stability (Chhetry et al., 2019; Kim et al., 2018).

### MoS_2_ film strain sensor for human health-related signals monitoring

**Figure 4:**
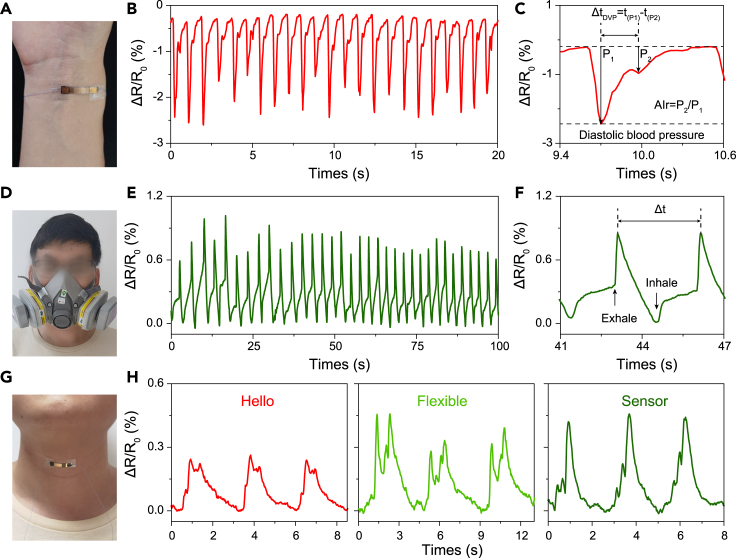
Application of the flexible MoS_2_ film strain sensor for health-related signals monitoring (A–C) Pulse signal monitoring: (A) photograph, (B) real time, and (C) enlarged single cycle of a wrist pulse signal of a 29-year-old healthy man. (D–F) Respiratory signal monitoring: (D) photograph, (E) real time, and (F) enlarged single cycle of a respiratory signal. (G and H) Vocal cord signal monitoring: (G) photograph and (H) real-time vibration signals of “hello,” “flexible,” and “sensor.”

Some health-related signals of the human body are inapparent, such as radial artery pressure, and real-time detection of these signals is vital for disease prevention (Li et al., 2020). The MoS_2_ film strain sensor, which has a low limit of detection and high GF, can be used for small-signal detection. Radial artery pressure (RAP) is used as an indicator in personal health and clinical diagnoses, and the shape of the RAP wave can assist doctors in diagnosing diseases such as arterial stiffness and high blood pressure (Zhang et al., 2019). Figures 4A–4C presents the MoS_2_ film strain sensor for RAP signal monitoring. Figure 4B shows the real-time signals of wrist pulses, and the pulse rate was 69 bpm, which was expected for a 29-year-old healthy man. The enlarged RAP wave is shown in Figure 4C, which also shows the incident wave (P_1_) and reflected wave (P_2_). Heart rate with time delay (Δt_DVP_ = t_P1_-t_P2_) and radial artery augmentation index (Al_r_ = P_2_/P_1_) could be extracted to be 277 ms and 42%, respectively, which were in the normal range for healthy males. Furthermore, the MoS_2_ film strain sensor was connected to a respirator to monitor breathing rate in real time, as shown in Figures 4D–4F. The breathing rate of the participant was 20 bpm (from Figure 4E), which was in the normal range for healthy adults (12–20 bpm). The enlarged view of a single respiratory cycle is shown in Figure 4F. The exhale and inhale are marked, and the duration of a respiratory cycle is 3.1 s. The MoS_2_ film strain sensor was also attached to a human throat (Figures 4G and 4H) to detect vocal cord vibrations when various words were spoken. Figure 4H shows the vibration signal for the words “hello,” “flexible,” and “sensor.” The reproducible and distinguishable signals for different words indicate that the MoS_2_ film stress sensor has potential in applications in intelligent speech recognition or speech correction exercises.

### Conclusions

In conclusion, we demonstrated a simple and efficient direct laser writing method to synthesize patterned, wafer-scale, and layered MoS_2_ film. The relevant characterization results indicated that the precursor (NH_4_)_2_MoS_4_ film could be effectively decomposed to form high-quality MoS_2_ film by a pulsed laser. Furthermore, the strain sensor was fabricated using the MoS_2_ film and showed a low detection limit (0.09%), high GF (1,118), and stability. Moreover, health-related signals, such as radial artery pressure, respiratory rate, and vocal cord vibrations, were monitored using the sensitive strain sensor. Our work contributes to the research on the development of large-area, high-quality 2D materials and adds to the diversification of flexible electronic materials by providing a new way to apply 2D TMD materials.

### Limitations of the study

This work focuses on synthesizing wafer-scale MoS_2_ film on a SiO_2_/Si substrate. However, given the laser processing range, preparing a larger MoS_2_ film with continuous fabrication needs further exploration. In addition, direct synthesis of a high-quality, large-area MoS_2_ film on a flexible substrate using a laser will greatly promote printed and flexible electronics development.

## STAR★Methods

### Key resources table

**Table:** 

REAGENT or RESOURCE	SOURCE	IDENTIFIER
**Chemicals, peptides, and recombinant proteins**		

N, N-Dimethylformamide	aladdin	CAS: 68-12-2
n-Butylamine	aladdin	CAS: 109-73-9
Ethanolamine	aladdin	CAS: 141-43-5
Ammonium tetrathiomolybdate	aladdin	CAS: 15060-55-6
PMMA	EM Resist Ltd	CAS: 9011-14-7
Potassium hydroxide	Sinopharm Chemical ReagentCo., Ltd	CAS: 1310-58-3

**Software and algorithms**		

3d Max	www.autodesk.com	2020
Adobe Photoshop	www.adobe.com	CC 2018
Adobe Illustrator	www.adobe.com	CC 2018
Origin	www.originlab.com	2018

**Other**		

Laser	Xi'an Langrui Technology Co., Ltd	LR-FIB-20/30
Optical microscopy	Nexcope	NM910
Confocal Raman Imaging	WITec	alpha300 R
Atomic Force Microscope	Asylum Research	Cypher S
X-ray Photoelectron Spectrometer Microprobe	Thermo Fisher Scientific	250Xi
Scanning Electron Microscope	Thermo Fisher Scientific	Apreo S
Thermal evaporation system	VNANO	VZZ-400
Source meter	Tektronix	Keithley 2450
Multimeter	Tektronix	Keithley 6500

### Resource availability

#### Lead contact

Further information and requests for resources and materials should be directed to and will be fulfilled by the lead contact, Prof. Xuewen Wang (iamxwwang@nwpu.edu.cn).

#### Materials availability

This study did not generate new unique reagents.

### Method details

#### Synthesis of the MoS_2_ film

The MoS_2_ film synthesis process is shown in Figure 1A. We dissolved 50 mg (NH_4_)_2_MoS_4_ in 20 mL DMF, 8 mL ethanolamine, and 8 mL n-butylamine mixed solution. The precursor solutions were magnetically stirred at 1000 rpm for 60 min and then stirred by ultrasound for an additional 60 min. A 285 nm SiO_2_/Si substrate was cleaned under air plasma at 100 W for 2 min. Then, the precursor solution was spin-coated on the SiO_2_/Si substrate with a gradient procedure of 500, 1,000, 1,500, and 2000 rpm for 10 s and 2500 rpm for 30 s. After, the SiO_2_/Si substrate was placed on a hot plate and baked at 150°C for 3min.

For the MoS_2_ film synthesis, the commercial laser marking machine (Xi'an langrui laser technology co., LTD) used a fiber laser with a wavelength of 1.06 *μ*m (spot size of ∼22 *μ*m). The prepared SiO_2_/Si substrate was placed under the field lens (110 mm^2^), and a programmable pattern was marked using EZCAD laser marking software. Different laser states can be obtained by adjusting the laser parameters, such as working distance (the distance between sample surface to the lens), laser power, pulse frequency, laser spacing, and writing speed. For a typical 5 mm × 10 mm area, the laser with a power density of 5.26 × 10^9^ W/m^2^ (laser fluence of 2.63 J/cm^2^) have been adopted under the optimized laser parameters as follows: working distance (170 mm), laser power (2 W), pulse frequency (200 kHz), laser spacing (2 μm), and writing speed (50 cm/s). After laser writing, the SiO_2_/Si substrate was washed in DMF at 130°C for 60 s to remove any residual precursor film. Lastly, the substrate was dried at 150°C, and the programmable MoS_2_ pattern remained on the SiO_2_/Si substrate.

#### Materials characterization

The morphology and microstructures of the MoS_2_ film were characterized using optical microscopy (Nexcope NMM910), Raman (WITEC Alpha 300R), AFM (Asylum Research Cypher S), XPS (Thermo SCIENTIFIC ESCALAB 250Xi) and SEM (FEI Apreo S). The Raman characterization was conducted using a 532 nm laser calibrated with a Raman peak of Si at 520 cm^−1^. The binding energy of the XPS results was corrected by assigning a value of 284.8 eV to the adventitious C 1s line.

#### MoS_2_ film strain sensor fabrication

The device fabrication process is shown in Figure 3A. The MoS_2_ film (with a typical area of 5 mm × 10 mm) was synthesized on an SiO_2_/Si substrate. Then, PMMA (950 A5) was spin-coated on the SiO_2_/Si substrate at a speed of 500 rpm for 5 s and 2000 rpm for 60 s. After baking at 120°C for 5 min, the SiO_2_/Si substrate was immersed in a hydroxide (KOH) solution at 90°C for 10 min. Then, PMMA was cleaned using deionized water and ready for device fabrication.

A 50 nm Au film was deposited on a 100 μm thickness PET substrate using the thermal evaporation system (VZZ-400, VNANO, China). An interdigital electrode with an electrode spacing of 100 μm was prepared using laser etching technology that had the same laser under the field lens of 70 mm^2^. The laser parameters are as follows: working distance (115 mm), laser power (2 W), pulse frequency (200 kHz), laser spacing (10 μm), and writing speed (1 m/s). PMMA with the MoS_2_ film was put on the PET to ensure the MoS_2_ film came into close contact with the electrode. After natural drying, PMMA was removed using acetone and cleaned using isopropyl alcohol, deionized water, and ethanol, respectively. After drying, the conductive carbon oil served as a bridge between the Au electrode and the test wire. The carbon oil was cured for 60 minutes at 60°C for further tests.

#### Electromechanical behavior characterization

A stepper motor was used to control the strain applied to the sensor, and a source meter (Keithley 2450, Tektronix) was used to measure the resistance of the sensor. The LabVIEW program was used for data acquisition. In addition, a multimeter (Keithley 6500, Tektronix) was used to test the health-related signals.

### Quantification and statistical analysis

Each value of sensor response (with standard deviation) and gauge factor shown in Figure 3C of the main manuscript corresponds to average value obtained from multiple measurements at a given ΔL from Figure 3B.

## Data Availability

• Data reported in this paper will be shared by the lead contact upon request. • No new code was generated during the course of this study. • Any additional information required to reanalyze the data reported in this paper is available from the lead contact upon request
