# Supplementary material for: Programmable patterned MoS2 film by direct laser writing for health-related signals monitoring
Source: iScience. 2021 Oct 16;24(11):103313. doi: 10.1016/j.isci.2021.103313 (PMC8564106; doi:10.1016/j.isci.2021.103313)
Supplement: Document S1. Figures S1 and S2 [file mmc1.pdf]

iScience, Volume *24*

Supplemental information

Programmable patterned MoS_2 film

by direct laser writing

for health-related signals monitoring

Manzhang Xu, Jiuwei Gao, Juncai Song, Hanxin Wang, Lu Zheng, Yuan Wei, Yongmin He, Xuewen Wang, and Wei Huang

Supplemental Figures:

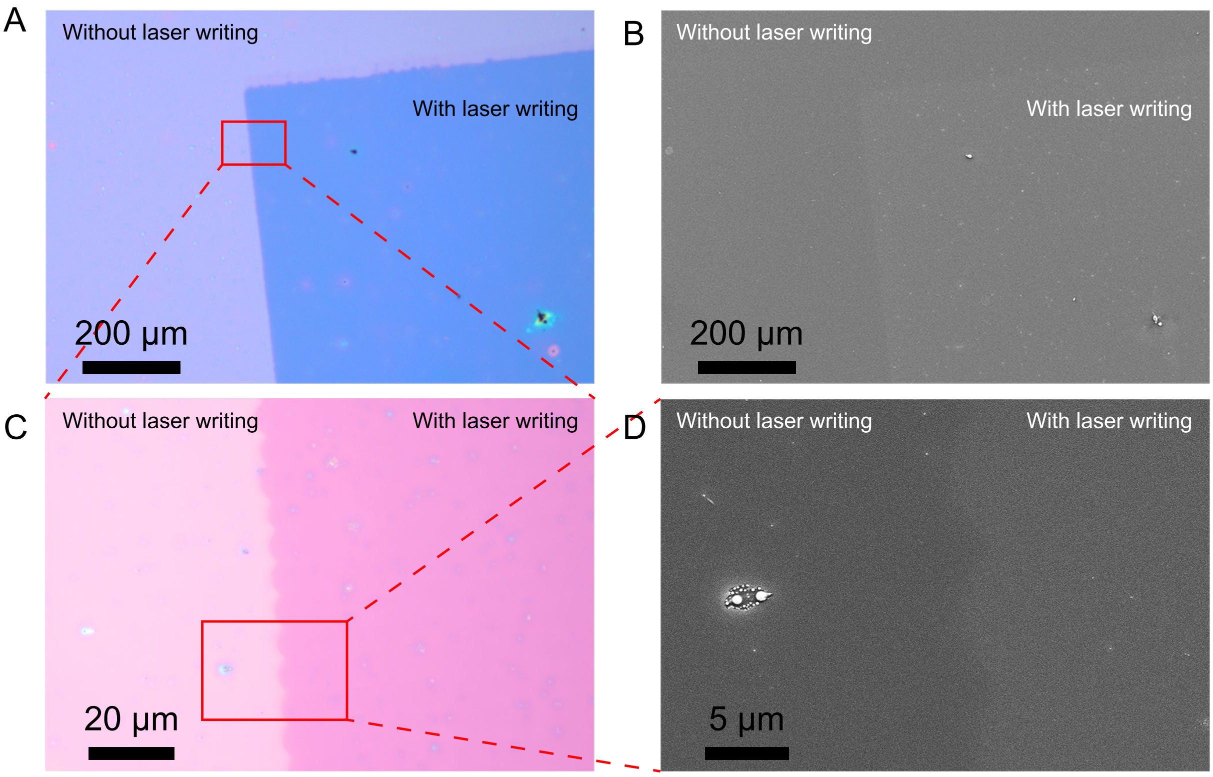

Figure S1. Optical and SEM images of MoS_2 film. (A) Optical image of and the corresponding SEM image of the MoS_2 film. (C) The enlarged optical image of the interface with and without laser writing in panel (A). (D) The enlarged SEM image of the interface with and without laser writing in panel (C). Related to Figure 2.

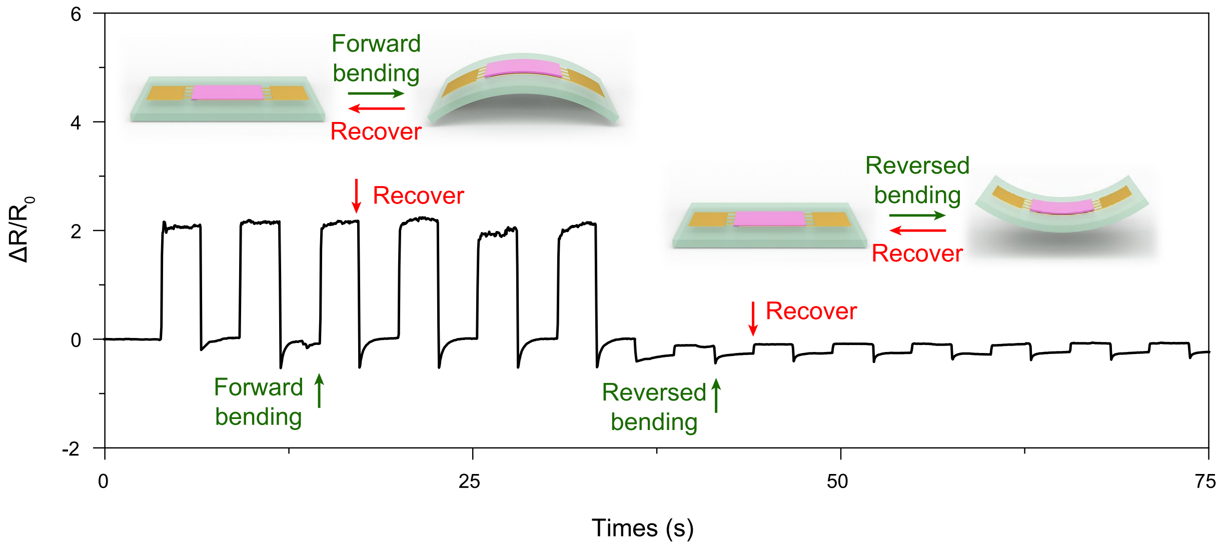

Figure S2. The flexible MoS_2 film strain sensor response under different bending modes. The resistance shows the opposite change under different bending. Related to Figure 3.
